# Comparison of oral and gut microbiome highlights the role of oral bacteria in systemic inflammation in HIV

**DOI:** 10.1128/msphere.00384-26

**Published:** 2026-10-05

**Authors:** Jennifer A. Fulcher, Katharine P. Newman, Bryan T. Pham, Fan Li, Grace D. Cho, Julie Elliott, Nicole H. Tobin, Steven Shoptaw, Pamina M. Gorbach, Grace M. Aldrovandi

**Affiliations:** 1 Division of Infectious Diseases, Department of Medicine, David Geffen School of Medicine at UCLA, Los Angeles, California, USA; 2 Division of Infectious Diseases, Department of Pediatrics, David Geffen School of Medicine at UCLA, Los Angeles, California, USA; 3 Department of Family Medicine, David Geffen School of Medicine at UCLA, Los Angeles, California, USA; 4 Department of Epidemiology, Fielding School of Public Health, University of California 5116 , Los Angeles, California, USA; Duke Human Vaccine Institute, Durham, North Carolina, USA

**Keywords:** human immunodeficiency virus, oral microbiome, human microbiome, inflammation

## Abstract

Chronic HIV-1 infection is associated with increased inflammation-related comorbidities, despite effective viral suppression with antiretroviral therapy. While the role of the gut microbiome in inflammation is well studied, the contribution of the oral microbiome remains less clear. This study investigates the relationship between the oral and gut microbiomes in driving systemic inflammation in persons with HIV. This cross-sectional study utilized archived samples from 198 participants (99 with HIV and 99 without HIV). Oral and gut microbiome composition was analyzed via 16S rRNA sequencing, and systemic inflammatory biomarkers were measured using multiplex assays. Bacterial inflammatory potential was assessed through *in vitro* co-culture and epithelial barrier permeability assays. The oral microbiome in HIV was characterized by increased *Veillonella*, *Capnocytophaga*, and *Megasphaera*, and several decreased genera including *Fusobacterium*. Using permutational multivariate analysis of variance, we found that the oral microbiome was a significant driver of cytokine variation in HIV compared to the gut microbiome and identified specific associations with oral *Veillonella* and *Megasphaera*. We found no differences in anti-*Veillonella parvula* serum IgG by HIV status, but IgG titers did correlate with microbial translocation markers sCD14 and LBP in HIV. *In vitro* studies demonstrated that *Veillonella parvula* increased oral epithelial barrier permeability and induced monocyte activation. These studies suggest that the oral microbiome, particularly *Veillonella parvula*, may contribute to systemic inflammation in HIV through mechanisms involving epithelial barrier disruption, oral translocation, and monocyte activation.

**IMPORTANCE:**

In HIV, persistent inflammation contributes to the elevated risk of cardiovascular disease, metabolic disorders, and other non-AIDS comorbidities. Alterations in the gut microbiome and resultant microbial translocation are contributors to this ongoing inflammation. Most microbiome research has focused on the gut compartment, though there is increasing appreciation for the role of the oral microbiome in chronic inflammatory diseases. Few studies have systematically compared the oral and gut compartments in HIV-associated inflammation. In this study, we identify *Veillonella parvula* as a plausible oral driver of epithelial barrier disruption and immune activation, expanding current understanding of microbial contributors to persistent inflammation in HIV.

## INTRODUCTION

Chronic HIV-1 infection is characterized by an accelerated risk of inflammation-related comorbidities. Despite the ability of antiretroviral therapy (ART) to effectively control viremia, persons living with HIV (PWH) have an increased risk of non-AIDS-related comorbidities, including cardiovascular disease and neurocognitive disease. While the cause is likely multifactorial, ample data support the role of inflammation in these processes (1–4), which is driven by mucosal barrier dysfunction and microbial translocation (5–7).

While the contribution of the gut microbiome to chronic inflammation in HIV is well described (5, 8), less is known about the role of the oral microbiome in inflammation in PWH. While several studies have found associations between HIV and oral dysbiosis (9–16), there is significant variation across studies due to variations in study design, sample size, and sampling techniques. Findings reported across multiple studies include alterations in *Veillonella*, *Fusobacterium*, *Rothia*, *Neisseria*, and *Streptococcus* (10, 11, 13–17). Several studies have found decreased alpha diversity over time during HIV (14, 17, 18). There are conflicting reports regarding the impact of ART, with some studies showing at least partial restoration of oral microbiome diversity after ART (10, 14, 18), but others showing little to no effect on diversity (17, 19).

Some studies have examined associations between the oral microbiome and inflammation in HIV. Yang et al. found that *Veillonella*, *Streptococcus*, and *Lactobacillus* were all associated with inflammatory biomarkers and were further associated with decreased lung function (15). At least one study simultaneously examined the oral and gut microbiomes in PWH and found that systemic inflammation biomarkers were inversely associated with saliva bacterial diversity, but not associated with the gut microbiome, suggesting a role for the oral microbiome in systemic inflammation in HIV (12).

The oral microbiome has been associated with multiple systemic diseases including cardiovascular disease, inflammatory bowel disease, rheumatoid arthritis, and dementia (20). Translocation of oral bacteria, primarily via the bloodstream, is one mechanism underlying these associations. Oral commensal bacteria, including *Fusobacterium nucleatum* and *Streptococcus* spp., have been identified in atherosclerotic plaques in coronary artery disease (21), suggesting a primary role for these bacteria in the bloodstream in cardiovascular disease. Ectopic colonization of the gastrointestinal tract by oral bacteria has also been implicated in disease development. Ectopic colonization of *Veillonella parvula* in the gut promoted development of colitis in a mouse model (22). We hypothesize that similar mechanisms contribute to inflammation in HIV. Here, we examine the relative contribution of the oral and gut microbiome to systemic inflammation in HIV and test the inflammatory potential of key bacteria.

## MATERIALS AND METHODS

### Study design and specimens

This cross-sectional study used archived specimens from the mSTUDY cohort (U01DA036267), a longitudinal cohort of men who have sex with men, with and without HIV, including half who actively use drugs based in Los Angeles, CA. Participants in the mSTUDY attend study visits approximately every 6 months, which consist of the collection of clinical data including laboratories, stored peripheral blood mononuclear cells (PBMCs), plasma, and serum, urine drug screen, stored rectal swab, stored saliva and oral rinse, and complete computer-assisted self-interview questionnaires detailing sexual behavior and substance use. The current study included blood, gut, and saliva samples from 198 mSTUDY participants selected based on paired blood, rectal swab, and saliva sample availability from the first study visit. mSTUDY samples used in this study were collected between 2014 and 2018. Rectal swabs were processed as described in prior studies (23, 24). Demographic and behavioral data were obtained from cohort study records. Saliva samples were collected by the passive drool method and then frozen at −80°C until processing in bulk.

### Oral microbiome profiling

Saliva processing and sequencing were performed by the University of California at San Diego Microbiome Core as previously described (25). Briefly, DNA extraction was performed using the MagMAX Microbiome Ultra Nucleic Acid Isolation Kit (Thermo Fisher Scientific) followed by sequencing of the V4 region of the 16S rRNA on the Illumina NovaSeq 6000 platform (2 × 150). Negative controls (extraction blanks and PCR negatives) and positive controls (ZymoBIOMICS Microbial Community Standard) were sequenced concurrently. Dada2 (v1.22.0), decontam (v1.14.0), and phyloseq (v1.38.0) were used for sequence inference, contaminant removal, and subsequent analyses. Taxonomic assignment was performed using the RDP training set version 18 and the “assignTaxonomy” and “addSpecies” functions from the “dada2” package with default parameters. Following all of these steps, a mean of 929,349 ± 333,428 reads per sample (Table S1) were used for further analysis, with no significant difference in depth by HIV status.

### Serum cytokine quantification

Serum samples from the same participants and study visits were used for cytokine analyses. Serum cytokines/biomarkers were measured using Meso Scale multiplex assays (Meso Scale Diagnostics) and ELISAs (R&D Systems) according to the manufacturer’s instructions. All samples were run in duplicate and processed in bulk to reduce inter-assay variability. Samples with a mean coefficient of variation (%CV) >20% were repeated. Values below the lower limit of quantification (LLOQ) were imputed as LLOQ/2.

### Bacterial culture and lipopolysaccharide purification

For *in vitro* experiments, representative species within each genus of interest were selected based on significance in the species-level analysis (Table S2) and commercial availability. *Fusobacterium nucleatum* subsp. *nucleatum* (ATCC 25586) and *Veillonella parvula* (strain ATCC 17742, Te 3) were purchased from the American Type Culture Collection (Manassas, VA, USA). Bacteria were grown under anaerobic conditions at 37°C in thioglycollate broth (cat# AS-801, Anaerobe Systems). Cultures were grown to an optical density at 600 nm (OD_600_) of 0.8–1.2, and then lipopolysaccharide (LPS) was purified using the iNtRON LPS Extraction Kit according to the manufacturer’s instructions (Boca Scientific). Purified LPS was quantified using the Pierce Chromogenic Endotoxin Quant Kit according to the manufacturer’s instructions (cat# A39553, Thermo Scientific).

### Anti-bacterial IgG ELISA

Bacteria were grown as described above for 24–48 h (OD_600_, 0.8–1.2), then cells were harvested by centrifugation at 10,000 × *g* for 10 min. The cell pellet was lysed using BugBuster Protein Extraction Reagent (Novagen), and the debris was removed by centrifugation at 20,000 × *g* for 10 min. The supernatant was collected, and the protein concentration was measured using the Pierce BCA Protein Assay. Ninety-six-well MaxiSorp plates (Nunc) were coated overnight with 50 μL of 0.5 mg/mL bacterial lysate. Plates were then washed with phosphate-buffered saline (PBS)/0.1% Tween (TPBS) and blocked with 3% milk/TPBS for 2 h at room temperature. Plates were washed with TPBS, and then the serum samples were added in serial 1:3 dilutions from 1:10 to 1:2,430 for 2 h at room temperature. After washing with TPBS, goat anti-human IgG-horseradish peroxidase (Bethyl Laboratories) was added at a 1:25,000 dilution for 1 h at room temperature. Plates were again washed, and 3,3′,5,5′-tetramethylbenzidine substrate was added, followed by stop solution for 20 min. Absorbance was measured at optical densities 450 and 650 using a plate reader (Tecan Spark). Endpoint titers were interpolated as described (26). In brief, the log-transformed dilution factor (e.g., 10 for 1:10 dilution) vs OD values was plotted on an *XY* plot. Pre-tested negative human serum was used to determine the positive cutoff, set at two standard deviations above the mean OD of negative serum. Endpoint titer was then interpolated using non-linear regression to determine the dilution (*X*) at that positive cutoff OD value. Interpolated endpoints were then compared by HIV serostatus using standard non-parametric tests (Mann-Whitney). All analyses were performed using Prism v10.4.2 (GraphPad).

### PBMC co-cultures

Bacteria were grown as described above for 24–48 h (OD_600_, 0.8–1.2), and then the cells were harvested by centrifugation at 10,000 × *g* for 10 min. Bacteria were resuspended in PBS and then heat-inactivated by incubation at 56°C for 30 min. Bacteria were counted by flow cytometry using the Bacteria Counting Kit with SYTO BC dye (cat# B7277, Invitrogen). PBMCs were co-cultured in Roswell Park Memorial Institute with 10% fetal bovine serum (FBS) overnight with 10 ng/mL LPS or inactivated bacteria at a bacteria-to-cell ratio of 25:1. Following co-culture, PBMCs were washed and stained for flow cytometry to examine T cell activation and monocyte subsets with the following antibodies: Pacific Blue anti-human CD3 (BioLegend, cat# 300431, clone UCHT1), APC-H7 anti-human CD8 (BD Pharmingen, cat# 560179, clone SK1), PE-Cy7 anti-human CD4 (BioLegend, cat# 344612, clone SK3), BB515 anti-human HLA-DR (BD Horizon, cat# 564516, clone G46-6), PE anti-human CD38 (BD Pharmingen, cat# 555460, clone HIT2), Brilliant Violet anti-human CD16 (BioLegend, cat# 302040, clone 3G8), APC anti-human CD14 (BioLegend, cat# 301808, clone M5E2), and PE/Dazzle 594 anti-human CD19 (BioLegend, cat# 302252, clone HIB19). Data were acquired on a BD FACSymphony A1 cell analyzer. Flow cytometry data were analyzed using FlowJo (v10.10.0). Gating strategy for analysis is depicted in Fig. S1.

### Oral epithelial barrier assay

TR146 buccal carcinoma epithelial cell line was purchased from Sigma-Aldrich (cat# 10032305) and grown to confluence in a transwell (Corning, 24-well, 0.4 μm pore) in Dulbecco’s modified Eagle medium (DMEM) with 10% FBS. Cells were plated at 24,000 cells/cm² in a transwell and cultured with media exchange every 2–3 days until confluent (total culture, 1–2 weeks). The integrity and barrier function of the confluent transwells were confirmed by measuring the transepithelial electrical resistance using a dual electrode connected to an epithelial volt/ohm meter (World Precision Instruments, Sarasota, FL, USA). Confluent transwells were then treated overnight with bacteria (25:1 bacterial-to-cell ratio) or LPS (10 ng/mL) or media in the apical chamber, and then the barrier function was assessed using dextran flux. After treatment with bacteria/LPS, the apical medium was removed and replaced with DMEM without FBS for 30 min at 37°C. Fluorescein isothiocyanate (FITC)-dextran (4 kDa; Sigma, cat# 46944) was added to the apical chamber (1 mg/mL), and then 100 μL media from the bottom chamber was sampled immediately and every 30 min for 2 h. Fluorescence was measured using a Tecan Spark fluorimeter (485 nm excitation, 525 nm emission). A control transwell with no cells was included as a measure of maximum flux, and all conditions were normalized as a percent of maximal flux for that time point (percent permeability). The percent barrier permeability for each condition was compared using Friedman tests. All analyses were performed using Prism v10.4.2 (GraphPad).

### Statistical analyses

Statistical analyses of the microbiome data were performed using the phyloseq (v1.38.0), vegan (v2.6.2), glmmTMB (v1.1.6), emmeans (v1.8.1.1), and twang (v2.5) packages within the R statistical environment (v4.1.2). Alpha diversity metrics were calculated using a rarefaction of 50,318 reads, and the differences were assessed using linear regression models with estimated marginal means. Permutational multivariate analysis of variance (PERMANOVA) was used to test for group-wise differences using Jensen-Shannon divergence.

To test for specific taxonomic differences between groups, we fit weighted negative binomial regression models for each taxon with weights derived from inverse probability of treatment weighting (IPTW). IPTW is a standard approach used to control for confounding variables in observational studies that utilizes propensity scores to create a pseudo-population in which there is no association between confounder and the treatment of interest. Propensity scores were calculated using gradient-boosted logistic regression with age, substance use, race/ethnicity, housing status, smoking, sexual behaviors, body mass index (BMI), and recent antibiotic exposure as selection variables, and HIV status as the treatment variable. An offset term was also applied based on the geometric mean of pairwise ratios normalization (27). Finally, models both with and without zero inflation were fit for each taxon, and the better model for each taxon was then selected according to the Akaike information criterion. All taxon-wise regression analyses were adjusted for multiple comparisons using the Benjamini-Hochberg false discovery rate (FDR) method, and results with an adjusted *P* value (*P*_adj_) <0.05 were accepted as significant.

To quantify the cytokine variation attributable to oral and gut microbiome profiles, we used a PERMANOVA-based approach (28). Specifically, we first computed the first 20 principal components for each microbiome compartment. PERMANOVA, as implemented in the “adonis2” function from the “vegan” R package using marginal tests and IPTW as described above, was then used to identify principal components that contributed significantly (*P* < 0.1) to the plasma cytokine profile. Finally, the percentage of cytokine variation attributable to each compartment was calculated as the sum of the variation attributable to all significant principal components from the given compartment. Integrative analysis of oral and gut microbiome profiles and plasma cytokines was performed using a multiblock sparse partial least squares discriminant analysis approach as implemented by the Data Integration Analysis for Biomarker Discovery using Latent Variable Approaches for Omics Studies (DIABLO) method in the mixOmics R package (v6.25.1). DIABLO constructs latent components by maximizing the covariances between data sets, while simultaneously balancing model discrimination and integration. Model tuning was performed using 10-fold cross-validation and 100 repeats. Based on the balanced error rate, a final model specification comprising two latent components was selected. A correlation cutoff of 0.6 was used to show links between the oral microbiome, gut microbiome, and plasma cytokine profiles.

For cytokines/biomarkers, values were log-transformed, and groups were compared using Kruskal-Wallis tests, with *P* values adjusted using the Benjamini-Hochberg FDR method, with *P*_adj_ < 0.05 accepted as significant (Fig. S2). Multiple linear regression analyses using least squares regression, including age, substance use (methamphetamine), and HIV category (negative, undetectable, suppressed, viremic) as predictors (Fig. S3), and Spearman correlations between cytokines/biomarkers and anti-bacterial IgG endpoints (Fig. 3) were performed as exploratory analyses. *P* values were adjusted using the Benjamini-Hochberg FDR method, with *P*_adj_ < 0.1 accepted as significant in these exploratory analyses. For PBMC stimulation experiments (Fig. 4), mixed-effects analysis was used to compare each treatment group to the control, with *post hoc* Dunnett’s multiple comparison test, and *P*_adj_ < 0.05 accepted as significant. For epithelial barrier assay experiments (Fig. 4), Friedman test was used to compare each treatment group to the control, with Dunn’s multiple comparison test, and *P*_adj_ < 0.05 accepted as significant. All analyses were performed using GraphPad Prism v10.4.2 unless otherwise noted.

## RESULTS

### Study design and sample characteristics

This study included samples from 198 individuals, including 99 persons with HIV (PWH) and 99 persons without HIV (PWOH). Participant demographics and characteristics are shown in Table 1. All participants were men who have sex with men. The group with HIV was older (34 vs 30 years; *P* < 0.001). Among those with HIV, the majority had well-controlled HIV (80.8% with viral load <200 copies/mL), and all participants had >200 CD4 T cells/mL. There were no significant differences by HIV status in terms of racial/ethnic groups, employment status, or body mass index. Sexual behavior was similar between those with and without HIV. The majority of participants did not smoke and had infrequent binge alcohol use, with no significant differences between those with and without HIV. Almost half of participants used methamphetamine, with no significant differences by HIV status in terms of frequency. More participants without HIV used cannabis than with HIV (53.5% vs 25.3%; *P* < 0.0001).

**TABLE 1:** Study participant demographics and characteristics^*a*^

Characteristics	Without HIV (*n* = 99)	With HIV (*n* = 99)	*P* value^*b*^
Age, mean (SD), median	30 (7.2), 28	34 (6.3), 35	<0.001
Race/ethnicity, *n* (%)			0.15
Black—non-Hispanic	43 (43.4)	30 (30.3)	
Hispanic	45 (45.5)	57 (57.6)	
Other—non-Hispanic	11 (11.1)	12 (12.1)	
Employment, *n* (%)			0.30
Full/part time	48 (48.5)	42 (42.4)	
Unemployed	35 (35.4)	42 (42.4)	
Disabled	6 (6.0)	10 (10.1)	
Student	10 (10.1)	5 (5.1)	
BMI, mean (SD), median	28 (6.5), 26	27 (5.6), 26	0.62
Pre-exposure prophylaxis, *n* (%)	26 (26.2)	NA	NA
Viral load, *n* (%); log mean (SD), median			NA
Undetectable (<20 copies/mL)	NA	42 (42.4); NA	
Suppressed (<200 copies/mL)	NA	38 (38.4); 3.4 (0.65), 2.9	
Unsuppressed (>200 copies/mL)	NA	19 (19.2); 9.1 (2.4), 9.8	
CD4 T cells, mean (SD), median	NA	667 (288), 638	NA
Antiretroviral therapy. *n* (%)			NA
NNRTI	NA	33 (35.9)	
PI	NA	17 (18.5)	
INSTI	NA	42 (45.6)	
Antibiotic use, *n* (%)	4 (4.0)	5 (5.1)	>0.99
No. of oral sex acts (30 days), mean (SD), median	12 (24), 5	14 (30), 5	0.89
No. of RAI acts (30 days), mean (SD), median	2.3 (5.1), 1	3.6 (6.9), 1	0.22
No, of sex partners (30 days), mean (SD), median	6.9 (8.5), 4	8.1 (9.3), 5	0.61
Methamphetamine use,^*c*^ *n* (%)			0.16
Daily	11 (11.1)	18 (18.2)	
Weekly	7 (7.1)	13 (13.1)	
Monthly/less	27 (27.3)	19 (19.2)	
Never	54 (54.5)	49 (49.5)	
Cannabis use, *n* (%)			<0.0001
Daily	11 (11.1)	0 (0)	
Weekly	8 (8.1)	6 (6.1)	
Monthly/less	34 (34.3)	19 (19.2)	
Never	46 (46.5)	74 (74.7)	
Smoking, *n* (%)			0.61
>1 pack/day	7 (7.0)	4 (4.0)	
<1 pack/day	37 (37.4)	36 (36.4)	
Nonsmoker	55 (55.6)	59 (59.6)	
Binge alcohol use,^*c*^ *n* (%)			0.14
Weekly	12 (12.1)	12 (12.1)	
Monthly/less	46 (46.5)	33 (33.3)	
Never	41 (41.4)	54 (54.6)	

^*a*^ SD, standard deviation; RAI, receptive anal intercourse; NA, not applicable.

^*b*^ *t*-tests or Mann-Whitney tests were used for continuous variables, and chi-squared tests were used for categorical variables.

^*c*^ Substance use data refer to past 6 months and were collected by self-report. Binge alcohol refers to >6 drinks at a time on more than one occasion.

### Oral microbiome composition differs between persons with and without HIV

The relative oral microbiome composition for each participant is shown in Fig. 1A. The most common genera in both groups include *Haemophilus*, *Veillonella*, *Streptococcus*, and *Prevotella*. There were no significant differences in alpha diversity between HIV groups (Fig. 1B); however, principal coordinates analysis did show marginal differences between those with HIV and without HIV (PERMANOVA, *P* = 0.09; Fig. 1C). Smoking and sexual behaviors were associated with overall microbiome composition (Table S3). We used generalized linear models to identify specific genera that distinguish the oral microbiome between PWH and PWOH. The oral microbiome in PWH showed decreased *Lactobacillus*, *Lautropia*, *Treponema*, *Oribacterium*, and *Fusobacterium*, as well as increased *Veillonella*, *Capnocytophaga*, and *Megasphaera* (Fig. 1D; Table S2) compared to PWOH.

**Fig 1:**
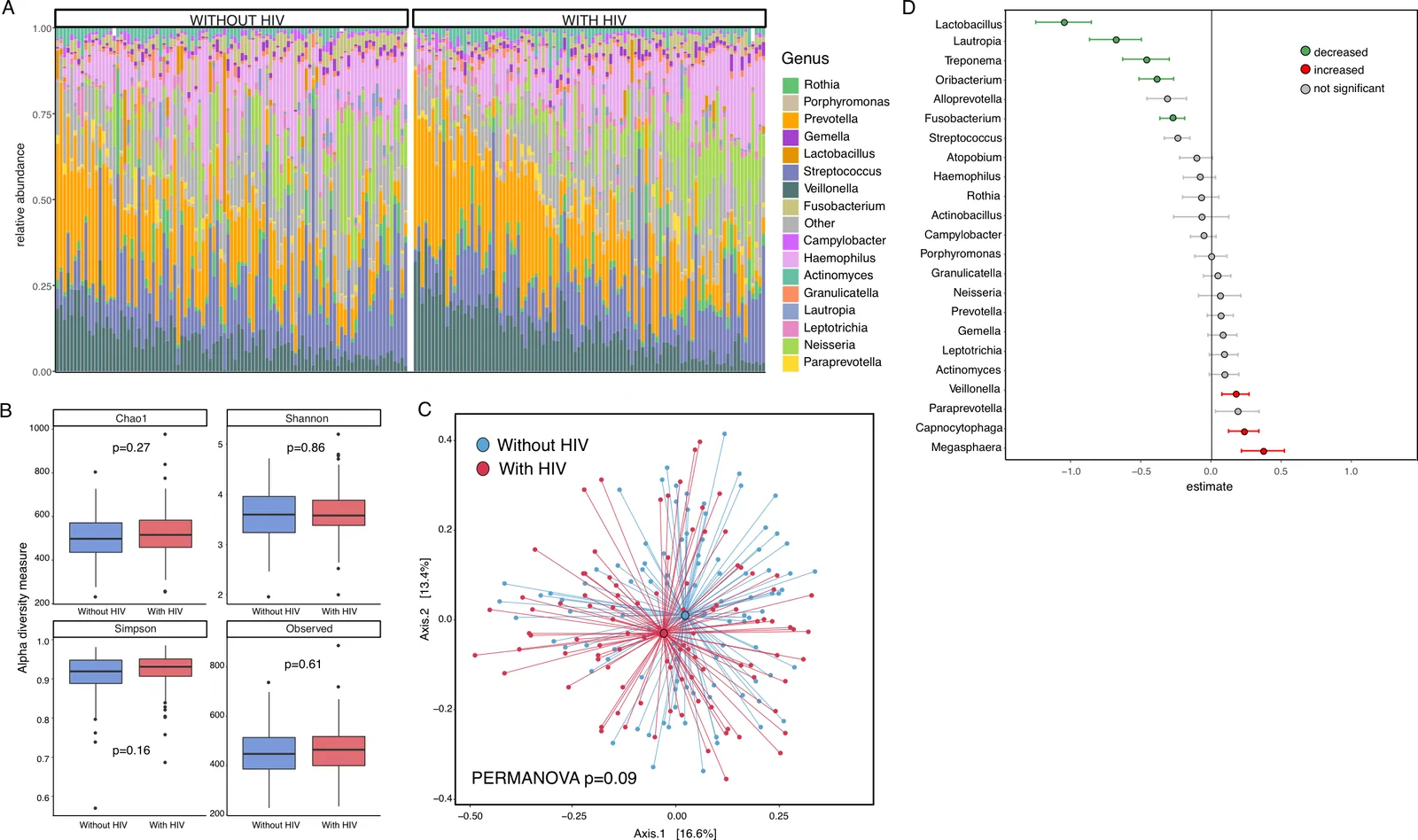
Oral microbiome composition differs in HIV. (**A**) Taxa bar plot showing oral microbiome composition for participants without HIV (left) and with HIV (right). Each bar represents individual participants, and colors show averaged relative abundance as a percentage of total bacterial sequences at the genus level. (**B**) Oral microbiome alpha diversity measures are not significantly different between persons with HIV (red) and without HIV (blue). (**C**) Principal coordinates analysis showing differences in oral microbiome between persons with HIV (red) and without HIV (blue). (**D**) Forest plot showing specific bacterial genera increased or decreased with HIV using zero-inflated negative binomial regression analysis. Green or red indicates associations that remained statistically significant (*P*_adj_ < 0.05) after FDR adjustment.

### Oral microbiome contributes significantly to systemic cytokine variation

Plasma cytokines and immune biomarkers were compared between PWH and PWOH. There were significant increases in several inflammatory cytokines/biomarkers in PWH, including TNF-α, IL-6, sCD163, sCD27, and CXCL10, independent of age, stimulant drug use, or viremia (Fig. S2 and S3). Using paired gut microbiome data from the same participants and study visits (23, 24), we quantified the contribution of each microbiome compartment on plasma cytokine variation using PERMANOVA. The gut and oral microbiome compartments together explained between 3% (IL-6) and 15.95% (sCD27) of the variation in plasma cytokine levels. Notably, sCD27, TNF-α, and IL-6 levels were driven by oral, but not gut microbiome compositions. In contrast, variation in IL-8 and CXCL10 levels was associated with both oral and gut microbiome compartments to approximately equal contributions, while IL-1β levels were driven more by the gut microbiome than the oral (Fig. 2A). We then used a supervised, N-integration approach called DIABLO to identify relationships between the two microbiome compartments and plasma cytokines. Briefly, DIABLO attempts to identify correlations between data layers while simultaneously optimizing prediction of an outcome variable. Using this approach, we found both oral *Veillonella* and *Megasphaera* to be positively correlated with IL-6 and FABP2 (Fig. 2B), suggesting a potential role in systemic inflammation. Additionally, both oral *Veillonella* and *Megasphaera* were negatively correlated with gut *Bacteroides* (Fig. 2B), which may suggest a role in gut dysbiosis. Finally, gut *Porphyromonas* was positively correlated with sCD27.

**Fig 2:**
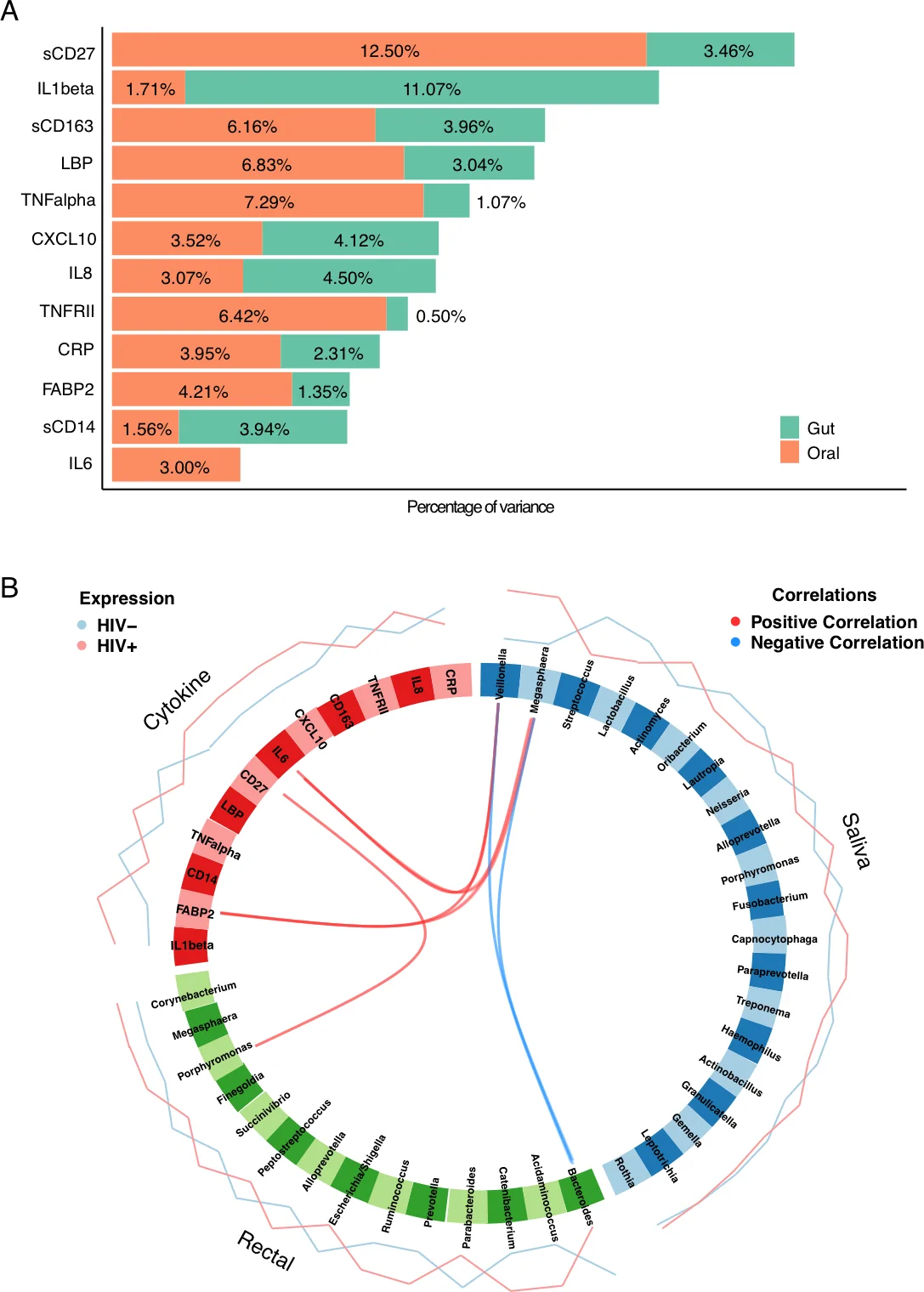
Relative contributions of oral and gut bacteria to cytokine variation in HIV. (**A**) Relative contribution of the gut (green) or oral (orange) microbiome to serum cytokine variation as determined by PERMANOVA. (**B**) Circos plot showing positive correlations (red) between *Porphyromonas* in the gut and *Veillonella* and *Megasphaera* in the saliva with inflammatory cytokines, and negative correlations (blue) with gut *Bacteroides*. Only correlations with *r* >0.6 are included.

### Plasma anti-bacterial IgG does not differ by HIV status

Given the positive correlation between *Veillonella* and *Megasphaera* with inflammatory cytokines, we hypothesized that systemic circulation of these bacteria could be a trigger for inflammation. To look for evidence of systemic circulation, we quantified anti-bacterial IgG in the plasma. For these two genera, we examined the species-level data (Table S2) and identified *Megasphaera micronuciformis* and *Veillonella parvula* as the most significant species in each genus. We focused on *Veillonella parvula* based on availability. For comparison, we also included *Fusobacterium nucleatum*, as it was significantly decreased in PWH (Table S2). There were no differences in anti-*Veillonella parvula* IgG or anti-*Fusobacterium nucleatum* IgG in PWH compared to PWOH (Fig. 3A).

**Fig 3:**
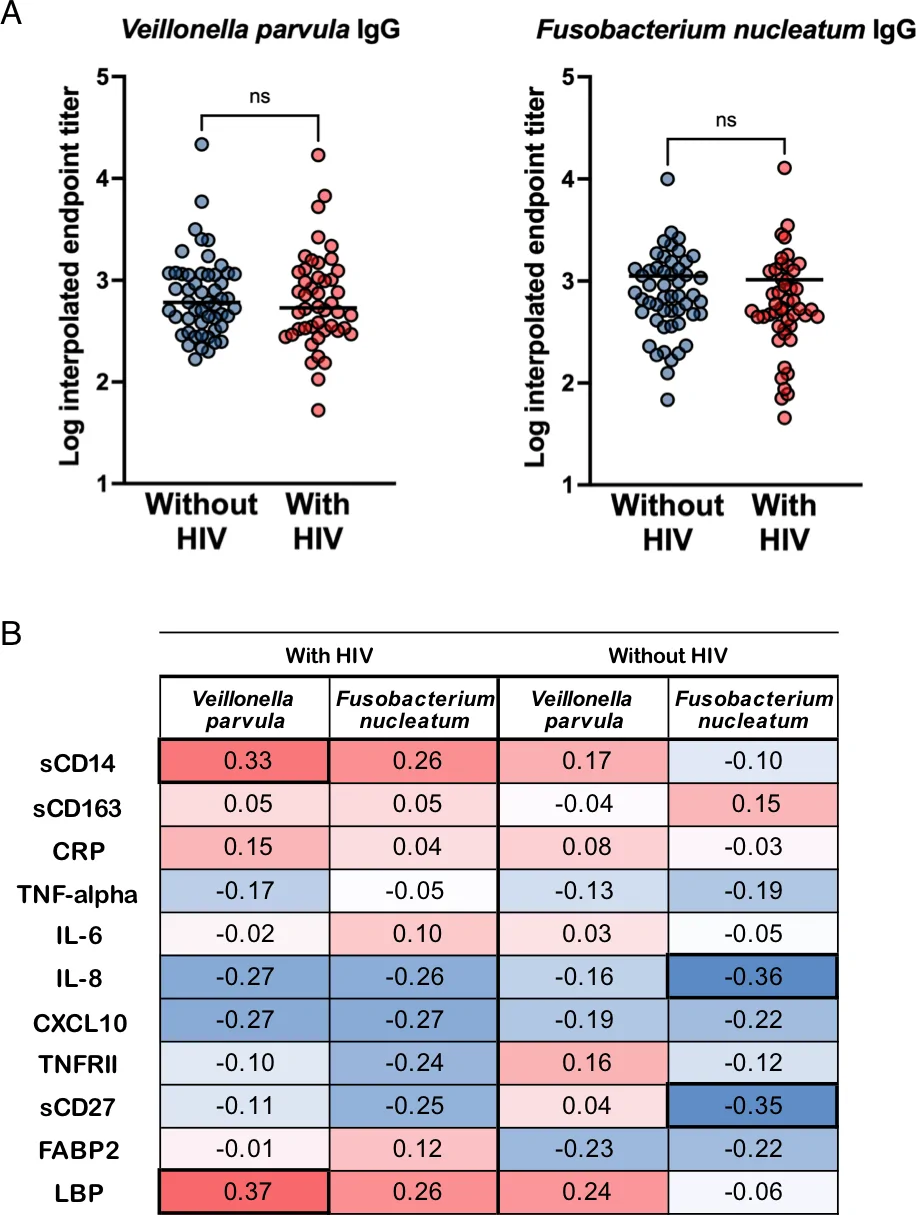
Anti-bacterial IgG endpoint titers correlate with microbial translocation markers in HIV. (**A**) Anti-*Veillonella parvula* or anti-*Fusobacterium nucleatum* IgG endpoint titers were measured in plasma by ELISA. Endpoint titers were interpolated from serial dilutions using negative pooled plasma as the cutoff. Titers were compared between HIV serogroups using Mann-Whitney tests, but not significant (ns). (**B**) Spearman correlation between inflammatory cytokines/biomarkers and anti-bacterial endpoint titers. The heatmap shows results separated by HIV status, with a red gradient indicating positive correlations and a blue gradient indicating negative correlations. Numbers denote Spearman rho values, with unadjusted *P* < 0.05 in bold.

### Anti-*Veillonella parvula* IgG is associated with microbial translocation markers in HIV

We next examined the relationship between anti-bacterial IgG and plasma cytokines using multiple linear regression with age and HIV status as covariates (Table S4). As expected, HIV was a significant predictor for several cytokines (sCD14, CD163, FABP2, IL-6, TNF-alpha, sCD27, and CXCL10). *Veillonella parvula* IgG was a significant predictor for sCD14 (β = 0.17, *P*_adj_ = 0.06) and LBP (β = 0.18, *P*_adj_ = 0.09), and *Fusobacterium nucleatum* IgG was a significant predictor for IL-8 (β = −0.14, *P*_adj_ = 0.09) and sCD27 (β = −0.11, *P*_adj_ = 0.04). Similarly, we examined Spearman correlations and found a positive correlation between *Veillonella parvula* IgG and microbial translocation markers sCD14 (*r* = 0.33, unadjusted *P* = 0.03) and LBP (*r* = 0.37, unadjusted *P* = 0.02), but only in PWH (Fig. 3B). *Fusobacterium nucleatum* IgG negatively correlated with IL-8 (*r* = −0.36, unadjusted *P* = 0.01) and sCD27 (*r* = −0.35, unadjusted *P* = 0.01), but only in PWOH. None of the correlations remained statistically significant after false discovery rate correction.

### *Veillonella parvula* increases oral epithelial barrier permeability

To further examine the hypothesis that *Veillonella parvula* oral translocation could contribute to systemic inflammation in HIV, we examined the effects of *Veillonella parvula*, both whole bacteria and isolated LPS, on oral epithelial barrier function. Using TR146 cells in a transwell system (29), we found that *Veillonella parvula* LPS, but not LPS from other bacteria, increased epithelial barrier permeability (Fig. 4A). *Veillonella parvula* LPS led to an average 44% barrier permeability (*P*_adj_ = 0.01 compared to untreated). In contrast, *Fusobacterium nucleatum* LPS (25%, *P*_adj_ > 0.99), *Escherichia coli* LPS (21%, *P*_adj_ > 0.99), and *Bacteroides fragilis* LPS (29%, *P*_adj_ = 0.18) did not significantly increase barrier permeability compared to untreated.

**Fig 4:**
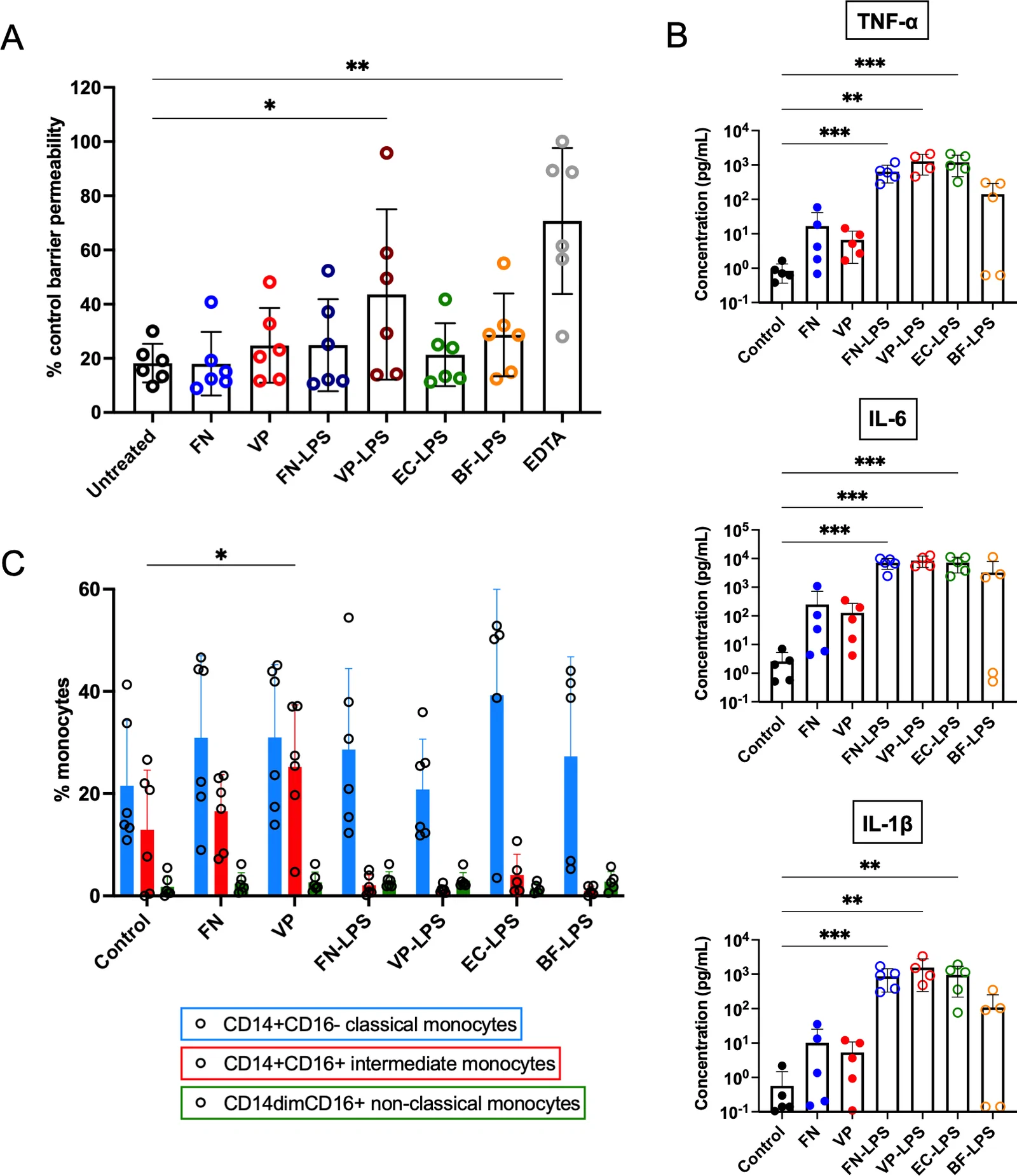
*Veillonella parvula* alters oral epithelial barrier function and induces PBMC responses. (**A**) FITC-dextran permeability assay using TR146 cells shows increased permeability following treatment with either heat-inactivated bacteria or isolated LPS from *Veillonella parvula* (VP). Permeability is expressed as a percent of transwell flux with no cells (set at 100%). Bars show the mean with error bars indicating standard deviation from six independent experiments. *, *P*_adj_ < 0.05; **, *P*_adj_ < 0.01 by Friedman tests with Dunn’s multiple comparison test. FN, *Fusobacterium nucleatum*; EC, *Escherichia coli*; and BF, *Bacteroides fragilis*. (**B**) Increased inflammatory cytokine production in supernatant from PBMC co-cultures with heat-inactivated bacteria or isolated LPS. Bars show the mean with error bars indicating standard deviation from five independent donors. **, *P*_adj_ < 0.01; ***, *P*_adj_ < 0.001 by mixed-effects analysis with *post hoc* Dunnett’s multiple comparison test. (**C**) Monocyte subsets as a percentage of total monocytes following co-culture with heat-inactivated bacteria or isolated LPS. Bars show the mean with error bars indicating standard deviation from five independent donors. *, *P*_adj_ < 0.05 by mixed-effects analysis with *post hoc* Dunnett’s multiple comparison test within each subset.

### Oral bacteria induce pro-inflammatory cytokine production and monocyte activation

To examine the capacity for *Veillonella parvula* to induce peripheral inflammation, we performed co-culture experiments with PBMCs. We found that LPS from *Veillonella parvula* significantly increased pro-inflammatory cytokine production, similar to LPS from *Fusobacterium nucleatum* and *Escherichia coli* (Fig. 4B). Co-culture with *Veillonella parvula* bacteria, but not LPS, increased the proportion of the inflammatory intermediate (CD14+CD16+) monocyte subset (Fig. 4C). There were no significant changes in other monocyte subsets or other cell populations, such as CD38+HLA-DR+ activated CD4+ or CD8+ T cells (data not shown).

## DISCUSSION

In this study, we identified several alterations to the oral microbiome in HIV, including increased *Veillonella*, *Capnocytophaga*, and *Megasphaera*. Using paired gut microbiome data, we also showed the significant contribution of the oral microbiome, particularly *Veillonella parvula*, to systemic inflammation in PWH.

Consistent with previous studies, our analysis revealed distinct differences in the oral microbiome composition between PWH and PWOH. We observed increased *Veillonella*, *Capnocytophaga*, and *Megasphaera* in HIV, along with a reduction in *Fusobacterium* among others (Fig. 1). Previous studies reported similar shifts in oral microbiota, specifically increased *Veillonella* and/or *Megasphaera* (9, 11, 13). Alterations in *Fusobacterium* have also been reported in HIV (12), though many other studies reported increases while we showed a relative decrease. Several factors can contribute to differences in specific findings across studies, including sampling methods and confounding factors. Our study used saliva samples, while others have more specific oral sampling such as plaque (12), which could account for some differences in results.

The positive correlations between *Veillonella parvula* IgG and microbial translocation markers (sCD14 and LBP) align with prior evidence suggesting systemic circulation of oral bacteria in inflammation-related disease states. In HIV, gut microbial translocation is a known cause of systemic inflammation (5, 30–32). To date, no studies have examined oral microbial translocation in HIV. While studies have correlated oral bacteria with systemic immune biomarkers (12, 15), none have investigated translocation mechanisms. In this study, we demonstrated that *Veillonella parvula* may alter oral epithelial barrier permeability, increase intermediate monocytes, and promote cytokine production similar to other bacteria (Fig. 4). Together, these findings suggest potential mechanisms by which *Veillonella parvula* could enhance systemic inflammation in HIV.

Another potential mechanism not directly tested in this study, but perhaps suggested by our data, is the translocation of oral bacteria to the gut as a driver of inflammation in HIV. In our integrative analysis, we found that *Porphyromonas*, a typical oral bacterium, in the gut compartment was positively associated with sCD27 (Fig. 2B). The emergence of oral bacteria in the gut has been observed in several inflammatory diseases (33), with mouse models showing that translocation of oral bacteria to the gut induces local and systemic inflammation (34–38). Further longitudinal studies are needed to investigate the temporal dynamics of oral to gut translocation and the impact on inflammation in HIV.

There are some limitations to consider with this study. First, there was only limited oral health data available to include in the analysis. We did not have dental exams or records available. As other studies have shown the importance of oral health in oral microbiome composition in HIV (39), this is an important limitation to note. Similarly, dietary information was not available, which is another notable limitation given the known impact of diet on the microbiome (40). Other confounders, including smoking, alcohol use, and substance use, were all addressed in the analyses using a propensity score-based weighting method. Next, our *in vitro* studies were limited to representative bacterial species/strains that are widely available, which may differ from clinical species/strains *in vivo*. Further studies could include similar experiments using isolates cultured from clinical samples to confirm these findings. Our study identified potentially important associations between the oral microbiome and inflammation in HIV, and suggests a plausible mechanism through *Veillonella parvula* barrier disruption, translocation, and monocyte activation.

## Data Availability

Data are available as supplemental files and/or deposited in accessible repositories. Sequencing data have been deposited in the NCBI BioProject database under the accession number PRJNA1291188. Metadata, cytokine data, and R scripts are available at https://github.com/Fulcher-Lab/mSTUDY-oral-microbiome.

## SUPPLEMENTAL MATERIAL

Supplemental figures msphere.00384-26-s0001.pdf Fig. S1 to S3.

Table S1 msphere.00384-26-s0002.xlsx Microbiome read counts by sample.

Table S2 msphere.00384-26-s0003.xlsx Negative binomial regression.

Table S3 msphere.00384-26-s0004.xlsx PERMANOVA.

Table S4 msphere.00384-26-s0005.xlsx Multiple linear regression results for each cytokine using age, HIV status, and anti-bacterial IgG titers as predictors.

ASM does not own the copyrights to Supplemental Material that may be linked to, or accessed through, an article. The authors have granted ASM a non-exclusive, world-wide license to publish the Supplemental Material files. Please contact the corresponding author directly for reuse.
