# Supplementary material for: Comparison of oral and gut microbiome highlights the role of oral bacteria in systemic inflammation in HIV
Source: mSphere. 2026 Oct 5;11(10):e00384-26. doi: 10.1128/msphere.00384-26 (PMC13711783; doi:10.1128/msphere.00384-26)
Supplement: Supplementary file 1 [file msphere.00384-26-s0001.pdf]

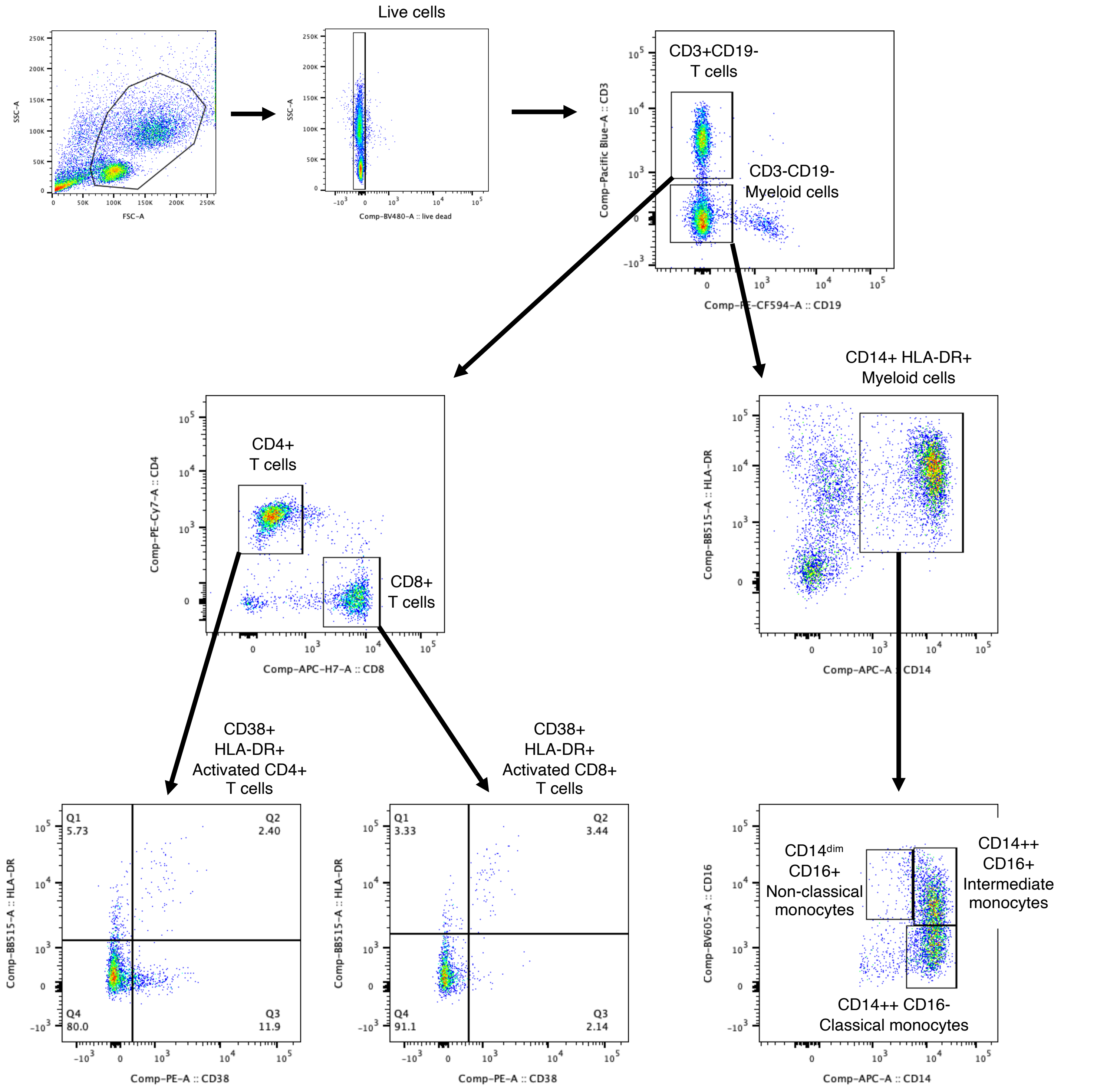

Supplemental Figure 1. Flow cytometry gating strategy for PBMC co-culture experiments. Flow cytometry gating strategy to examine T cell activation and monocyte subsets following PBMC co-cultures with bacteria and/or LPS. Cells were initially gated on forward and side scatter (top left) then live cells gated using dye exclusion. The live cell population was then gated into T cells (CD3+CD19-) and non-T/non-B cells or myeloid cells (CD3-CD19-). The T cell population was then gated into CD4+ and CD8+ T cell subsets then activation examined within these populations based on CD38 and HLA-DR expression (bottom left plots). The myeloid cell population was then gated on CD14+HLA-DR+ population for monocytes then subsets examined based on CD14 and CD16 expression (bottom right plot). Percentages of each population of interest were determined in FlowJo (v10.10.1) and used for statistical analysis as described.

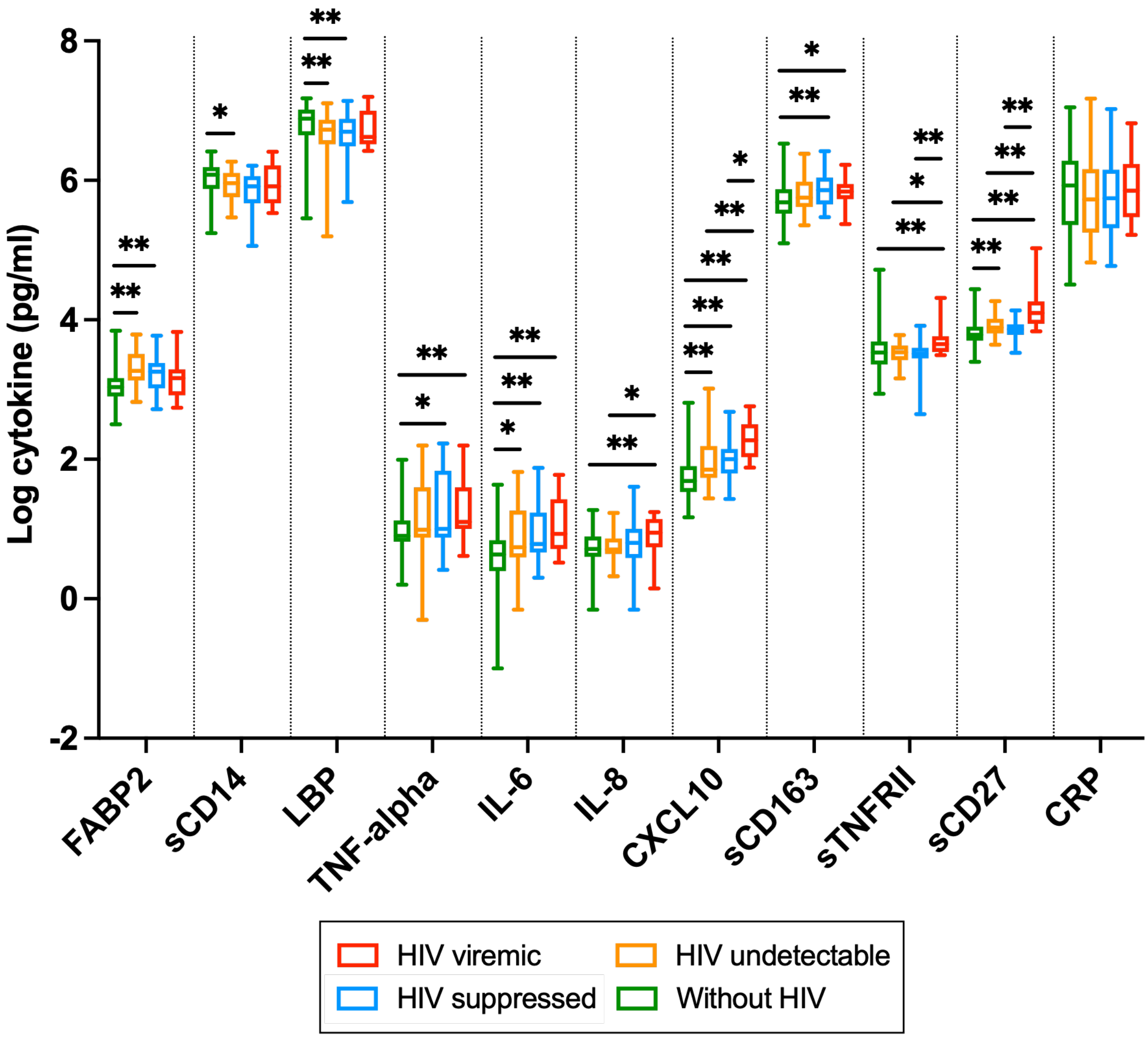

Supplemental Figure 2. Plasma cytokines/biomarkers by HIV viremia groups. Middle lines denote median with whiskers min/max. *$p_{adj}<0.05$; **$p_{adj}<0.01$; *** $p_{adj}<0.001$ by Kruskal-Wallis tests with Benjamini-Hochberg false discovery rate (FDR) correction.

		Estimate	Standard Error	Adj p-value
**FABP2**	Intercept	2.9	0.085	**<0.001**
	Age	0.0058	0.0027	**0.083**
	Stimulant Use	-0.0031	0.036	0.99
	HIV undetectable	0.18	0.042	**0.008**
	HIV suppressed	0.16	0.075	0.10
	HIV viremic	0.062	0.064	0.55
**sCD14**	Intercept	6.0	0.085	**<0.001**
	Age	-0.00059	0.0027	0.98
	Stimulant Use	0.11	0.037	**0.017**
	HIV undetectable	-0.090	0.043	0.10
	HIV suppressed	-0.13	0.076	0.19
	HIV viremic	-0.087	0.065	0.35
**LBP**	Intercept	6.8	0.10	**<0.001**
	Age	-0.0026	0.0033	0.63
	Stimulant Use	0.049	0.044	0.48
	HIV undetectable	-0.12	0.051	**0.063**
	HIV suppressed	-0.028	0.091	0.95
	HIV viremic	-0.059	0.078	0.63
**sCD163**	Intercept	5.8	0.083	**<0.001**
	Age	-0.0024	0.0026	0.58
	Stimulant Use	-0.0053	0.036	0.98
	HIV undetectable	0.11	0.041	**0.027**
	HIV suppressed	0.35	0.074	**0.0083**
	HIV viremic	0.17	0.063	**0.027**
**CXCL10**	Intercept	1.6	0.10	**<0.001**
	Age	0.0059	0.0033	0.16
	Stimulant Use	0.0090	0.044	0.98
	HIV undetectable	0.18	0.051	**0.0083**
	HIV suppressed	0.23	0.091	**0.033**
	HIV viremic	0.53	0.078	**0.0083**
**TNF-alpha**	Intercept	0.87	0.15	**<0.001**
	Age	0.0027	0.0048	0.77
	Stimulant Use	0.10	0.065	0.24
	HIV undetectable	0.16	0.076	**0.083**
	HIV suppressed	0.27	0.14	0.12
	HIV viremic	0.30	0.12	**0.033**
**IL-6**	Intercept	0.46	0.16	**0.004**
	Age	0.0046	0.0050	0.58
	Stimulant Use	0.017	0.068	0.98
	HIV undetectable	0.24	0.079	**0.017**
	HIV suppressed	0.43	0.14	**0.014**
	HIV viremic	0.40	0.12	**0.0083**
**IL-8**	Intercept	0.65	0.084	**<0.001**
	Age	0.0022	0.0027	0.61
	Stimulant Use	0.016	0.036	0.88
	HIV undetectable	0.0061	0.042	0.98
	HIV suppressed	0.091	0.075	0.43
	HIV viremic	0.18	0.064	**0.027**
**sCD27**	Intercept	3.8	0.057	**<0.001**
	Age	-5.2e-005	0.0018	0.99
	Stimulant Use	0.00030	0.025	0.99
	HIV undetectable	0.082	0.029	**0.025**
	HIV suppressed	0.090	0.051	0.17
	HIV viremic	0.34	0.044	**0.0083**
**CRP**	Intercept	5.9	0.20	**<0.001**
	Age	-0.00050	0.0062	0.99
	Stimulant Use	-0.0020	0.084	0.99
	HIV undetectable	-0.10	0.098	0.54
	HIV suppressed	0.061	0.17	0.94
	HIV viremic	0.12	0.15	0.63

Supplemental Figure 3. Regression analysis of immune cytokines/biomarkers in people with and without HIV. Multiple linear regression was performed using least squares regression with age, simulant use (yes/no) and HIV viremia category (undetectable, suppressed <200 copies/ml, or viremic >200 copies/ml) as predictors. P-values adjusted using Benjamini-Hochberg false discovery rate (FDR) with adj p-value <0.1 considered significant in this exploratory analysis.
